# 

**Published:** 1898-10

**Authors:** 


### CONTENTS.

### COMMUNICATIONS.

Leucoplakia.—Abstract ... 451
Preliminary and Graduating Requirements—The Logical Standard 465
Management of the Pulpless Teeth ... 468
Preparation of Borders of Cavities for Filling ... 475
A Method of Handling Alveolar Pyorrhea.—Abstract ... 478
The Effect of Heat on Dentine ... 481

### PROCEEDINGS.

National Dental Association, Omaha, 1898 ... 483

### SELECTIONS.

The Widow of the late Sir Morell Mackenzie ... 490
Pride Before A Fall ... 490
Orthoform—A Local Anesthetic for Open Wounds, Burns, Ulcers, Etc. 491
Official Privileges Extended to Women Physicians ... 495
Divorce and Suicide ... 496

### EDITORIAL.

The National Association of Dental Faculties ... 496
National Dental Association ... 497
A Very Proper Thing ... 498
Ohio State Dental Society ... 499

### OBITUARY.

Died Lately in Holland ... 499

